# Supplementary material for: The deubiquitylating enzyme USP15 regulates homologous recombination repair and cancer cell response to PARP inhibitors
Source: Nat Commun. 2019 Mar 15;10:1224. doi: 10.1038/s41467-019-09232-8 (PMC6420636; doi:10.1038/s41467-019-09232-8)

Supplementary Information

The deubiquitylating enzyme USP15 regulates homologous recombination repair and cancer cell response to PARP inhibitors

Peng et al

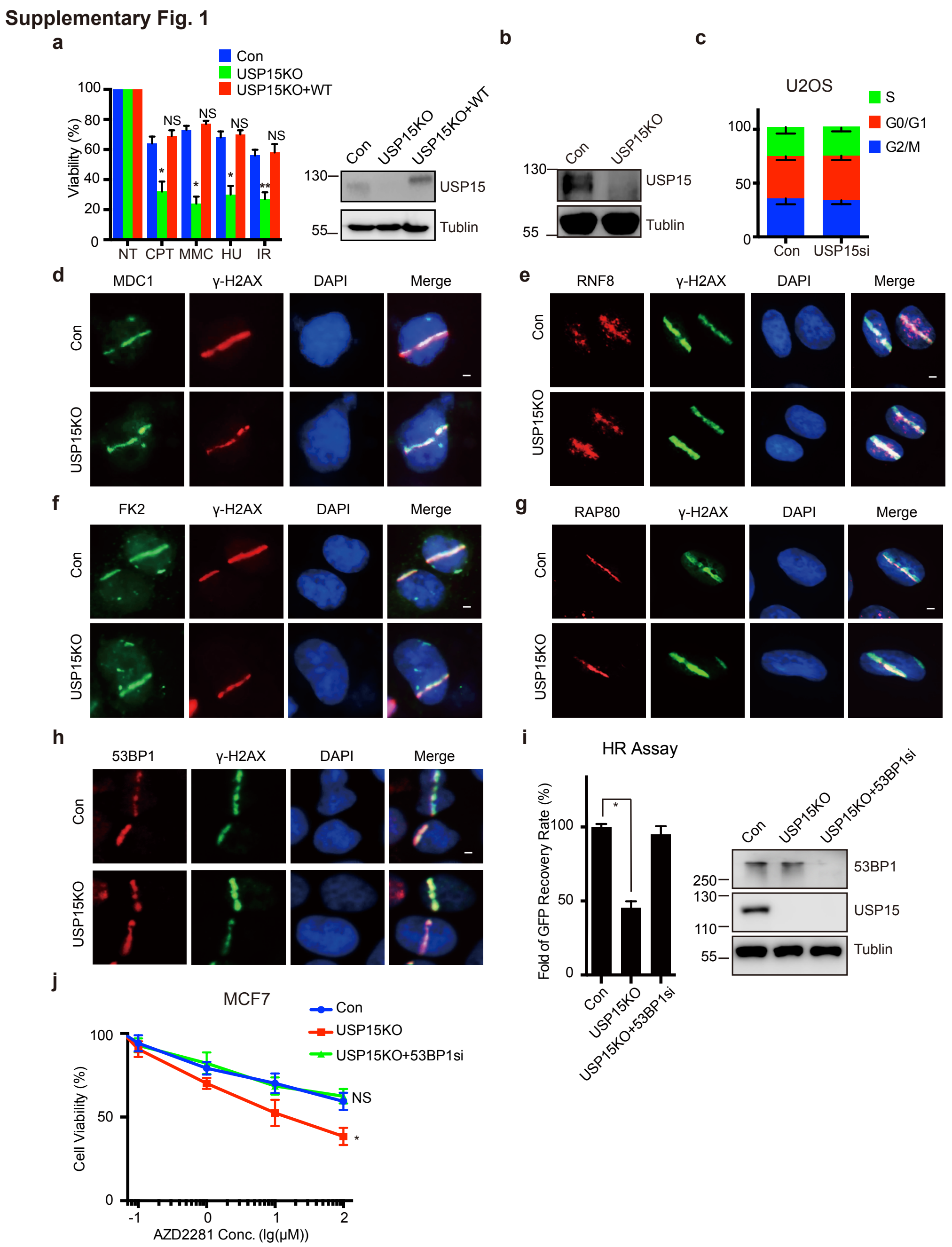

Supplementary Fig. 1 | USP15 regulates DNA damage repair, related to Fig. 1. a, Viability analysis of USP15 depledted MCF7 cells compared to wild type cells, after exposing to indicated DNA damage-inducing or replication stress-inducing agents (Left panel). Data are presented as mean ± SEM of three independent experiments. Two-way ANOVA, *P<0.01; **P<0.001. Expression of USP15 was examined (Right panel). **b**, USP15 knockout efficiency were verified in CRISPR mediated USP15 knockout U2OS cells, correlated to Fig. 1a. **c**, Cell cycle analysis of wild type or USP15 knockdown U2OS cells. **d-h**, Wild type or USP15 depleted U2OS cells were subject to micro-irradiation as described in Method section. Then cells were fixed and subject to immunofluorescence with the indicated antibodies. Representative images of MDC1 (**d**), RNF8 (**e**), FK2

(**f**), RAP80 (**g**), and 53BP1 (**h**) accumulation at sites of Laser induced DNA damage are shown. Scale bar: 10 µm. **i**, USP15 depleted U2OS DR-GFP cells were transfected with 53BP1 siRNA and HR efficiency were determined. Data are presented as mean ± SD of three independent experiments. Two tailed students' t test, *P<0.01. **j**, USP15 depleted MCF7 cells were transfected with 53BP1 siRNA. Cells response to PARP inhibitor (AZD2281) were analyzed by MTS assay. Data are presented as mean ± SEM of three independent experiments, two-way ANOVA, *P<0.01.

Supplementary Fig. 2

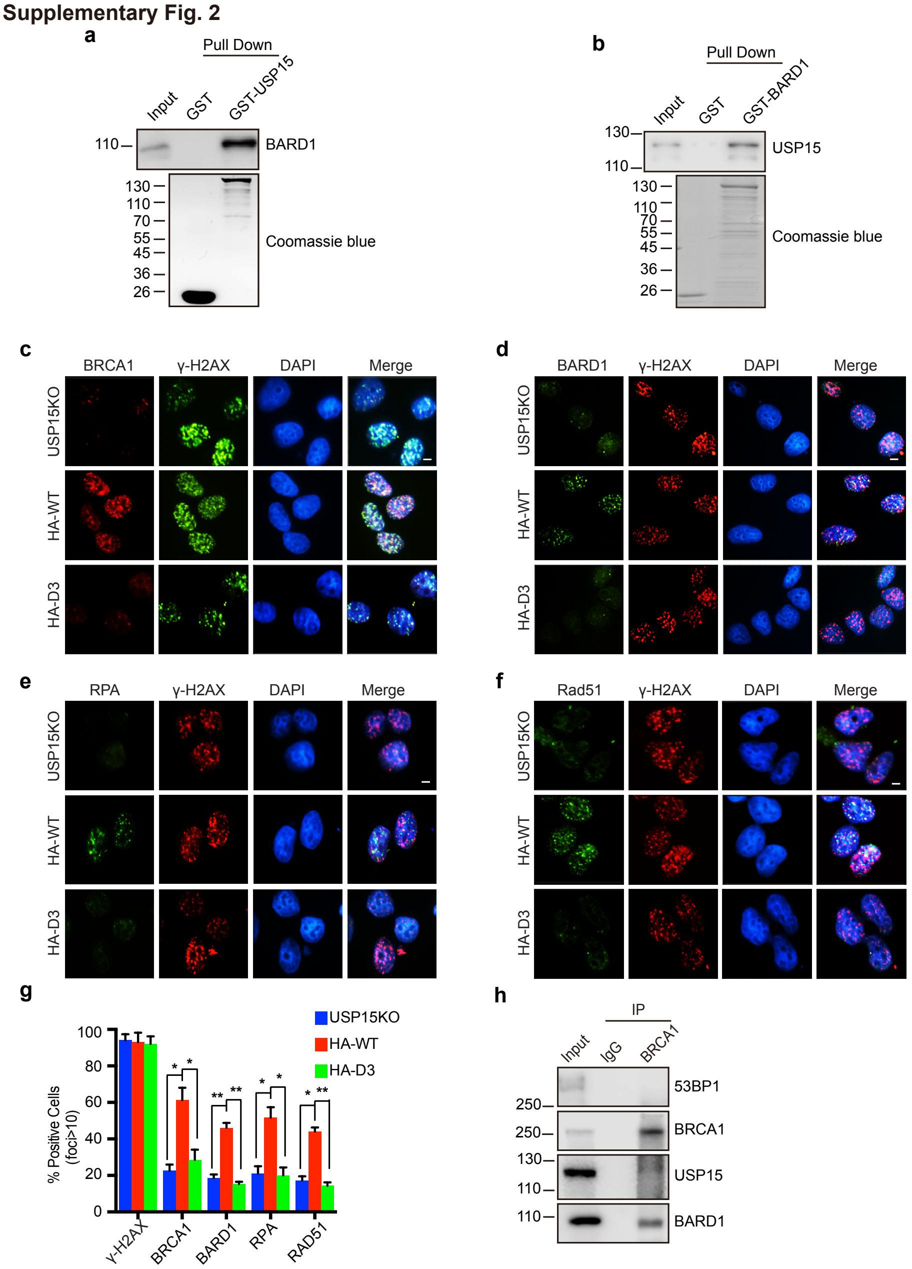

Supplementary Fig. 2 | USP15 interacts with BARD1 and this interaction is essential for USP15's functions in HR, related to Fig. 2. a, **b**, GST-pull-down assay of USP15 or BARD1 using the indicated proteins expressed in bacteria. **c-f**, USP15 depleted U2OS cells were reconstituted with HA-USP15 WT or D3 mutant. The indicated DNA damage factors' foci formation were examined following IR (4Gy) treatment and 2 hrs' recovery. Representative images of BRCA1 foci (**c**), BARD1 foci (**d**), RPA foci (**e**), and RAD51 foci (**f**) are shown. Scale bar: 10 μm. **g,** Quantification results of **c-f** were presented as the mean ± SD of three independent experiments. 100 cells were counted in each experiment. Two tailed students' t test, *P<0.05, **P<0.01. **h**, Endogenous

immunoprecipitation (IP) with anti-BRCA1 antibody were performed and samples were detected by western blot with indicated antibodies.

Supplementary Fig. 3

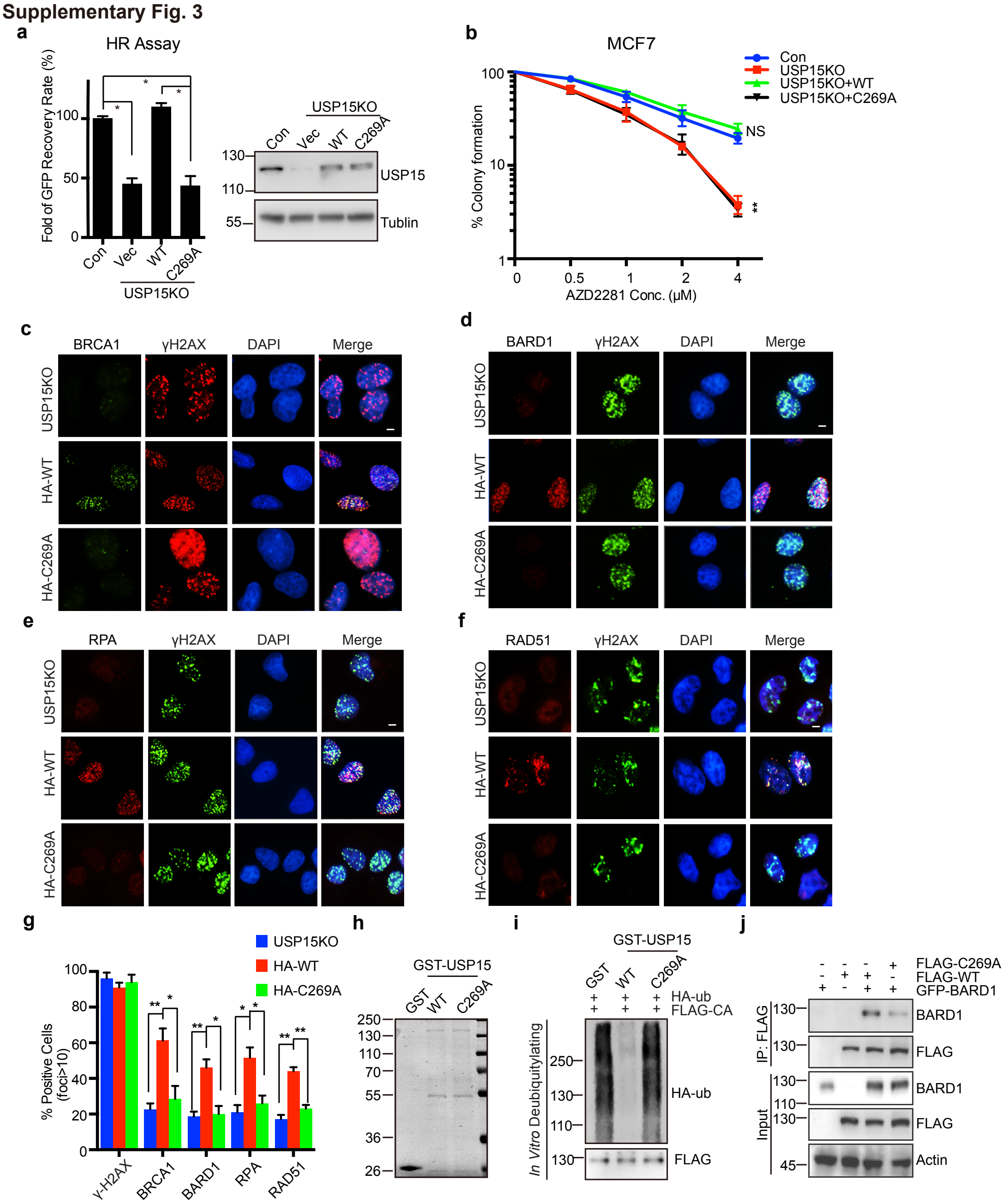

Supplementary Fig. 3 | The deubiquitinating enzyme activity of USP15 is essential for its functions in HR, related to Fig. 3. a, USP15 depleted U2OS DR-GFP cells were reconstituted with HA-USP15 WT or C269A mutant and were subject to HR reporter assay. Data are presented as mean ± SD of three independent experiments. Two tailed students' t test, *P<0.01. **b**, USP15 depleted MCF7 cells were reconstituted with USP15 WT or C269A mutant. Cells response to PARP inhibitor (AZD2281) were performed by colony formation assay. Data are presented as mean ± SEM of three independent experiments, two-way ANOVA, *P<0.01. **c-g**, USP15 depleted U2OS cells were reconstituted with USP15 WT or C269A mutant; and subjected to IR (4Gy). Representative images of BRCA1 foci (**c**), BARD1 foci (**d**), RPA foci (**e**), RAD51 foci (**f**) are shown. Scale bar:

10 μm. **g**, Quantification of **c-f** are presented as mean ± SD of at least three independent experiments, 100 cells were counted in each experiment. Two tailed students' t test, *$P<0.05$, **$P<0.01$. **h**, GST-USP15 WT or GST-USP15 C269A proteins were purified from BL21 *E. Coli*. Proteins were separated by SDS gel and were shown by coomassie blue staining. **i**, USP15 counteract its own ubiquitination. Ubiquitinated USP15CA proteins were purified from 293T cells and *in vitro* deubiquitination assay were performed. Samples were subject to immunoblot with the indicated antibodies. **j**, USP15 catalytically inactive mutant shows weaker binding with BARD1. 293T cells were transfected with indicated plasmids, then cells were lysed and were immunoprecipitated with FLAG M2 beads. Samples were subject to immunoblot with the indicated antibodies.

Supplementary Fig. 4

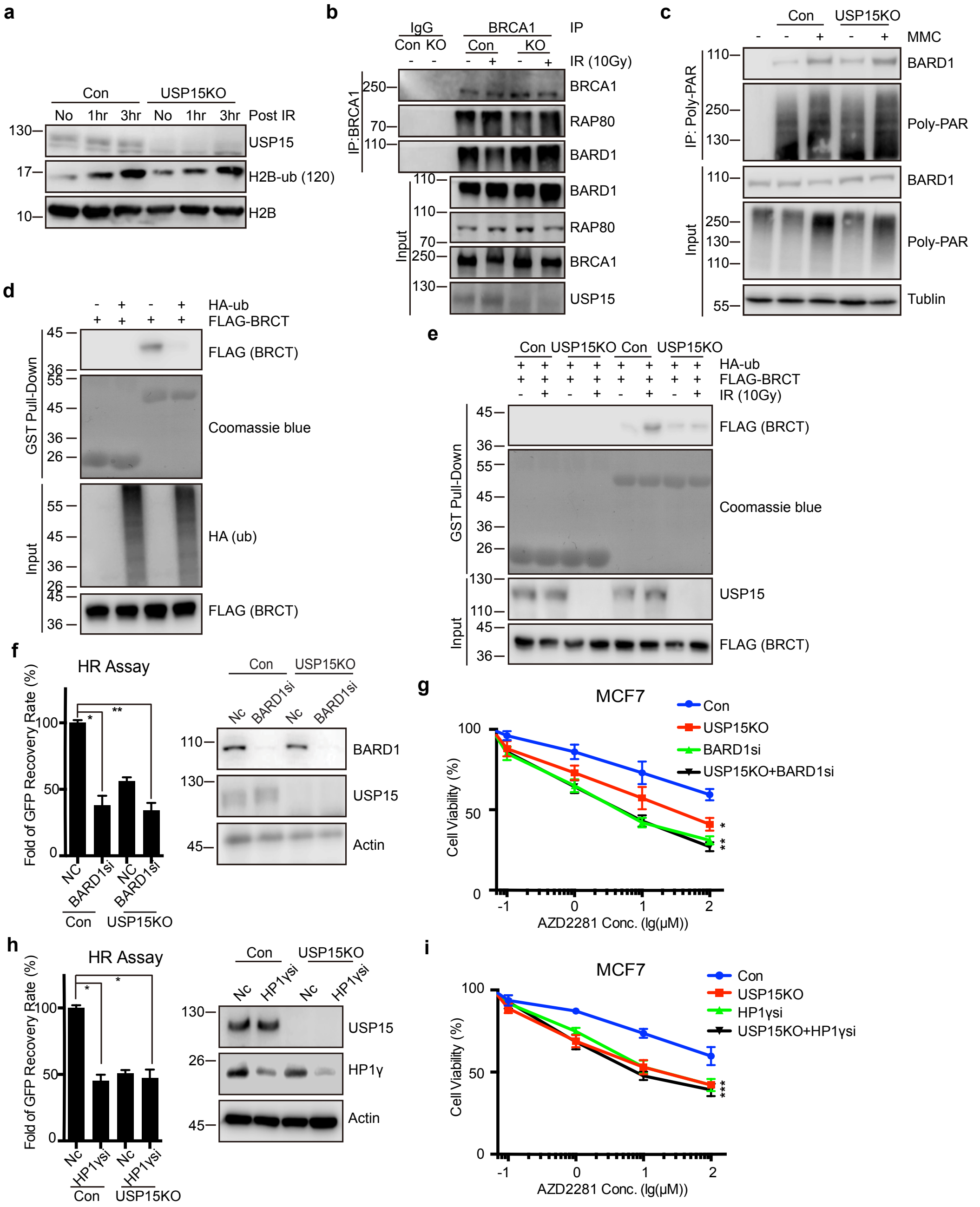

Supplementary Fig. 4 | USP15 deubiquitinates BARD1 and regulates BARD1 recruitment by HP1γ, related to Fig. 3. a, Immunoblot analysis of H2B monoubiquitination at indicated times following irradiation (10Gy). Cells were lysed with 2N HCl and neutralized by NaOH, samples were detected by Western Blot with indicated antibodies. **b**, Co-immunoprecipitation (Co-IP) assay were performed to detect BRCA1-A complex assemble in USP15 depleted cells upon DNA damage. Cells with indicated treatment were lysed and BRCA1 were immunoprecipitated. Samples were detected by Western Blot with indicated antibodies. **c**, Co-IP assay were performed to detect BARD1 and PAR interaction in USP15 depleted cells. Cells were lysed and immunoprecipitated with anti-poly-PAR antibody after indicated treatment for 24 hrs. Samples were detected

by Western Blot with the indicated antibodies. **d**, Interaction between HP1γ and ubiquitinated BARD1 BRCT domain were detected by GST-pull-down assay. **e**, USP15 facilitates BARD1-HP1γ interaction upon DNA damage. Cells with indicated treatment were lysed under denaturing conditions; and were subject to GST-pull-down assay. Samples were detected by Western Blot with indicated antibodies. **f**, Analysis of HR efficiency in USP15 depleted cells transfected with Scramble siRNA or BARD1 siRNA. Data are presented as mean ± SD of three independent experiments, two tailed students' t test, *P<0.05, **P<0.01. **g**, Wild type or USP15 depleted MCF7 cells were transfected with Scramble siRNA or BARD1 siRNA, and cells response to PARP inhibitor were investigated by the MTS assay. Data are presented as mean ± SEM of three independent experiments, two-way ANOVA, *P<0.01. **h**, Analysis of HR efficiency in USP15 depleted cells transfected with Scramble siRNA or HP1γ siRNA. Data are presented as mean ± SD of three independent experiments, two tailed students' t test, *P<0.01. **i,** Wild type or USP15 depleted MCF7 cells were transfected with Scramble siRNA or HP1γ siRNA, and cells response to PARP inhibitor was investigated by the MTS assay. Data are presented as mean ± SEM of three independent experiments. Two-way ANOVA, *P<0.01.

Supplementary Fig. 5

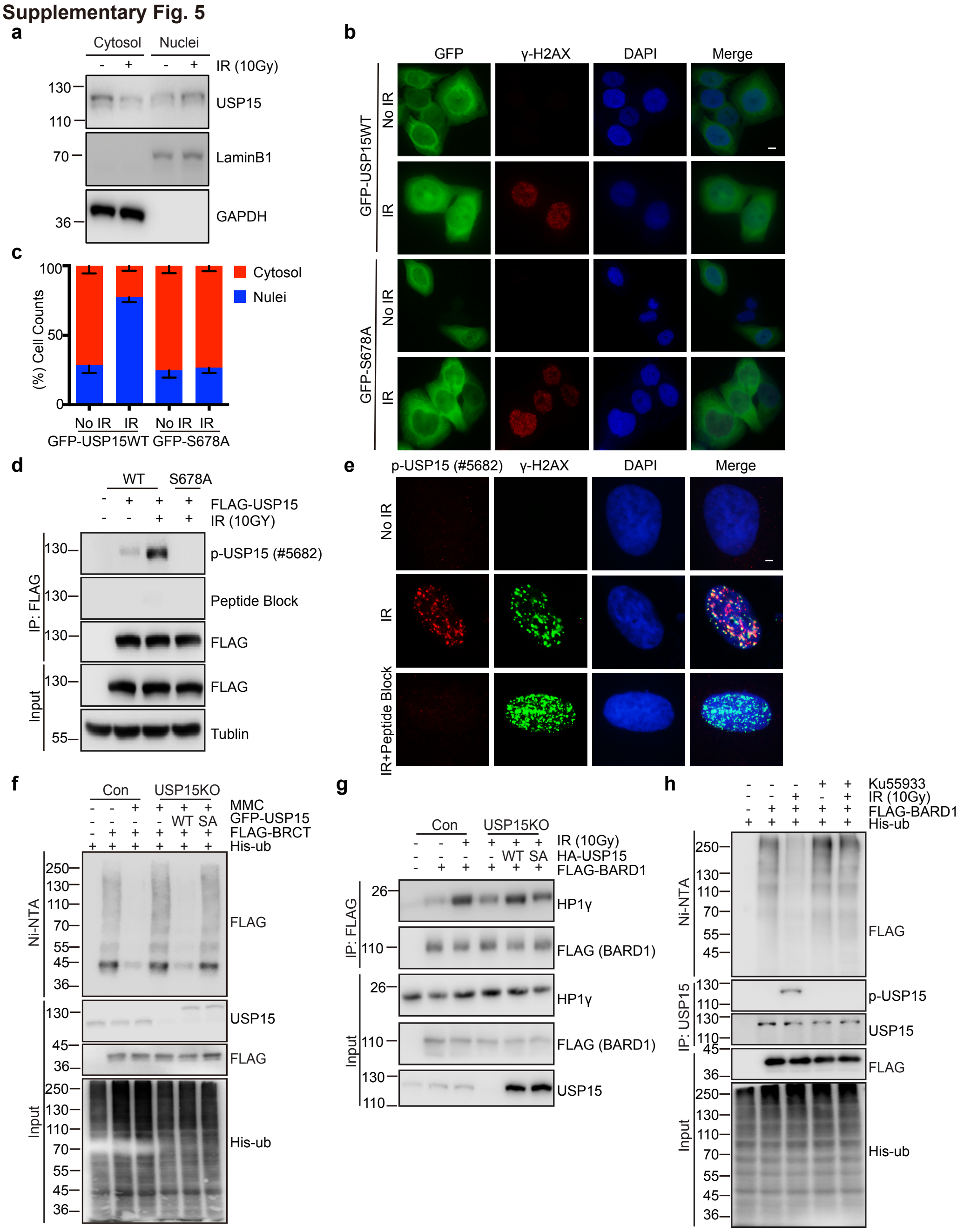

Supplementary Fig. 5 | USP15 translocate from cytoplasm to nuclear upon DNA damage, related to Fig. 4. a, 293T cells were treated with or without IR (10Gy), 3 hrs later, cytoplasm and nuclei were separated, and were subject to immunoblot with indicated antibodies. **b**, USP15 translocate to nuclear upon DNA damage. U2OS cells stably expressing GFP-USP15 WT or S678A were treated with or without IR (10Gy), 3 hrs later, cells were fixed, and immunofluorescence were performed with indicated antibodies. Scale bar: 10 μm. **c**, Quantification of **b** are shown. **d**, USP15 depleted 293T cells were reconstituted with USP15 WT or USP15 S678A mutant and were treated as indicated. Then exogenous USP15 protein were immunoprecipitated and phosphorylation of USP15 were detected with the phospho-specific antibody. The specificity of the

antibody was further validated by peptide blocking. **e**, U2OS cells were treated with or without IR (4Gy) and were immunostained with the phospho-specific antibody. The specificity of the antibody was further validated by peptide blocking. Scale bar: 5 μm. **f**, USP15 depleted 293T cells reconstituted with GFP-USP15 WT or S678A mutant were treated as indicated for 24 hrs, cells were then lysed and ubiquitinated proteins were pull down by Ni-NTA. Samples were detected by Western Blot with indicated antibodies. **g**, USP15 depleted 293T cells reconstituted with GFP-USP15 WT or S678A mutant were treated as indicated, 3 hrs later, cells were immunoprecipitated with FLAG M2 beads. Samples were subject to immunoblot with the indicated antibodies. **h**, 293T cells transfected with indicated plasmids were treated with or without ATM inhibitor Ku55933 (10μM). 24 hrs later cells were treated with or without IR (10Gy) as indicated and were allowed to recover for 2 hrs. Half of the cells were subject to denatured deubiquitination assay, while half of the cells were immunoprecipitated with anti-USP15 antibody. Samples were detected by Western Blot with indicated antibodies.

Supplementary Fig. 6

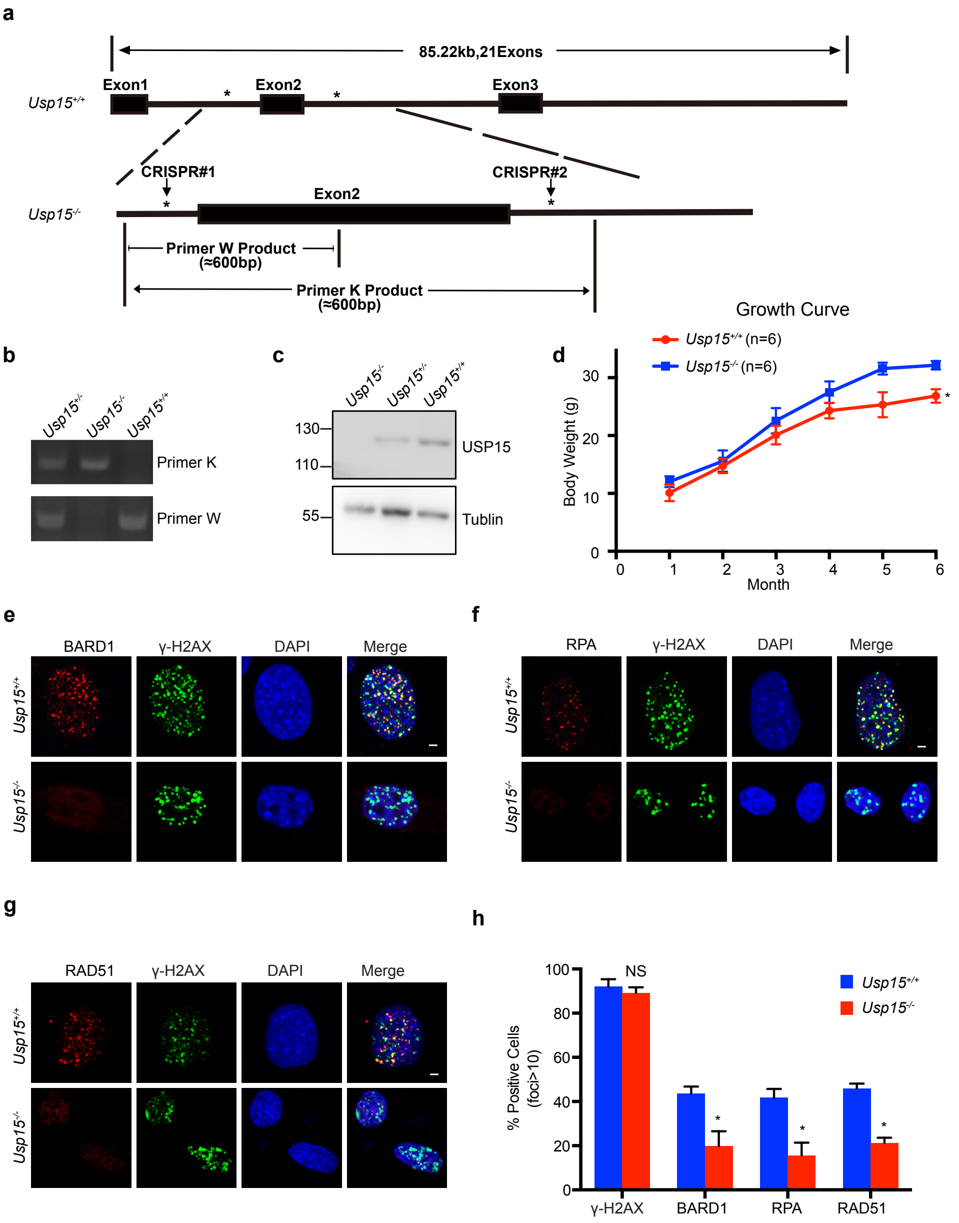

Supplementary Fig. 6 | USP15 knockout mice show genome instability, related to Fig. 6. a, Schematic representation of generates of USP15 KO mice. **b**, USP15 KO mice were verified by genotyping. Mice genome DNA was extracted from tails of new born pulps. Genotyping were performed using indicated primers, as described in method section. **c**, Mice protein was extracted from ear tissues of 4-week-old mice. Samples were separated by SDS-gel and were blotted with indicated antibodies. **d**, 6 pairs of *Usp15*$^{+/+}$ or *Usp15*$^{-/-}$ littermates were randomly picked and mouse weight was measured monthly. Growth curves are shown up to 6 months. **e-g**, *Usp15*$^{+/+}$ or *Usp15*$^{-/-}$ MEFs were treated with IR (4Gy), 3 hrs later, cells were fixed and were subject to immunofluorescence with indicated antibodies. Representative images of BARD1 foci (**e**), RPA foci (**f**),

RAD51 foci (**g**) are shown. **h**, Quantification of **e**-**g** are presented as mean ± SD of three independent experiments, 100 cells were counted in each experiment. Two tailed students' t test, *$P<0.05$. Scale bar: 5 μm.

Supplementary Fig. 7

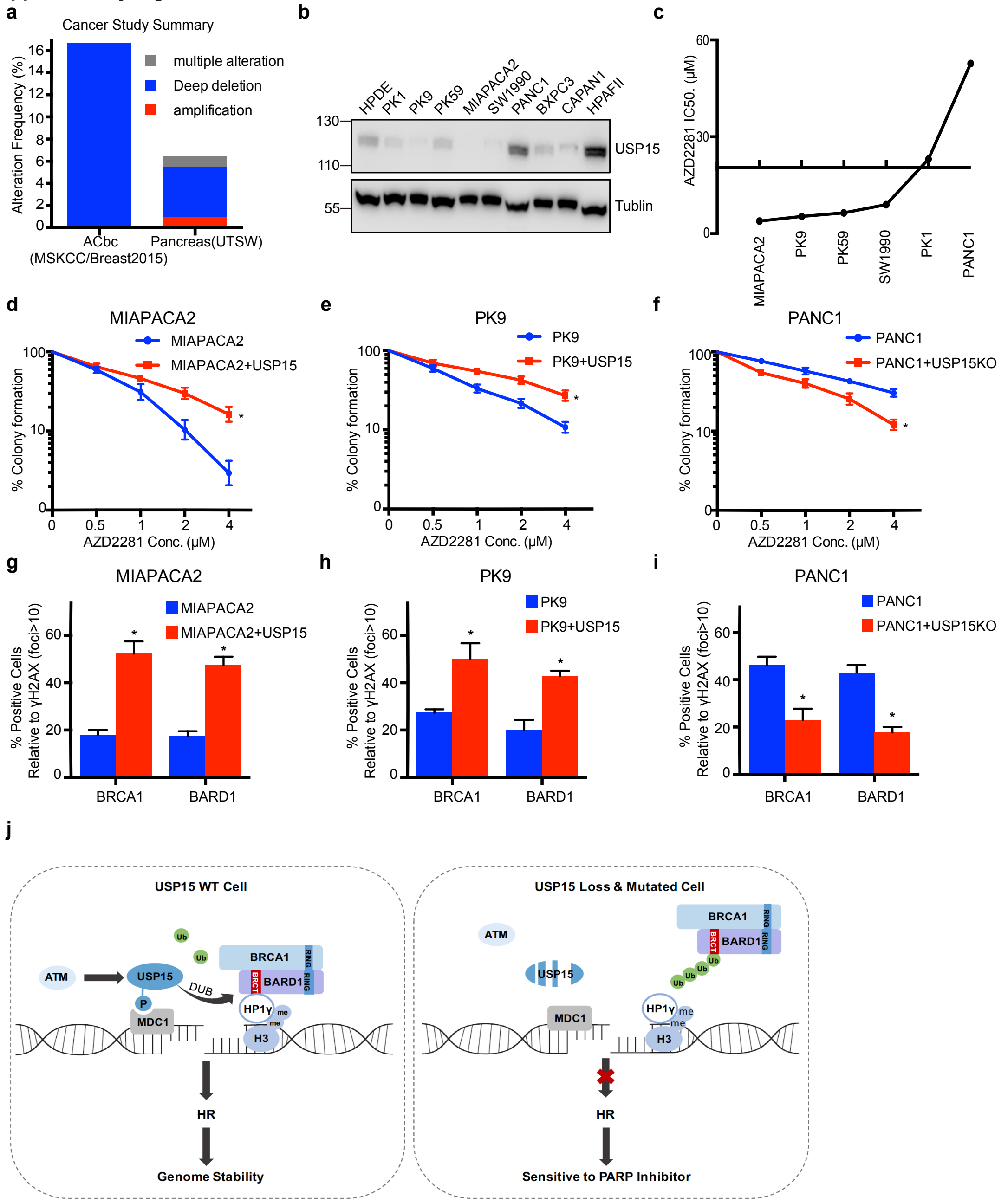

Supplementary Fig. 7 | USP15 regulates pancreatic cancer cell response to PARP inhibitor. a, USP15 amplification or deletion in cancers was analyzed based on the public database (http://www.cbioportal.org). **b**, Cell lysates from pancreatic cancer cell lines were blotted with USP15 antibodies. Lysates from normal pancreatic (HPDE6-E6E7c7) epithelial cells were used as controls. **c**, Pancreatic cancer cells response to PARP inhibitor (AZD2281) was investigated by MTS assay. Then IC50 value were measured and plotted on the graph. **d**, **e**, MIAPACA2 or PK9 cells were stably overexpressed with USP15, and sensitivity to PARP inhibitor (AZD2281) were then determined by colony formation assay. Data are presented as mean ± SEM of three independent experiments. **f**, PANC1 cells were infected with the indicated viruses, and

sensitivity to PARP inhibitor (AZD2281) were then determined by colony formation assay. Data are presented as mean ± SEM of three independent experiments. The data from **d-f** were analyzed by two-way ANOVA, *P<0.05, **P<0.01. **g**, **h**, MIAPACA2 or PK9 cells were stably overexpressed with USP15, and were treated with AZD2281 (10μM) for 8hr. Then BRCA1/BARD1 foci formation in these cells were measured. Data are presented as mean ± SD of three independent experiments. **i,** PANC1 cells were infected with indicated viruses, and were treated with AZD2281 (10μM) for 8hr. Then BRCA1/BARD1 foci formation in these cells were measured. Data are presented as mean ± SD of three independent experiments. The data from **g-i** were analyzed by two-way ANOVA, *P<0.05, **P<0.01. **j**, A working model demonstrating how USP15 regulates HR and cancer cell response to PARP inhibitor.

Supplementary Fig. 8 Full blots related to Fig. 1, Fig. 2, Fig. 3, Fig.S1, Fig. S2.

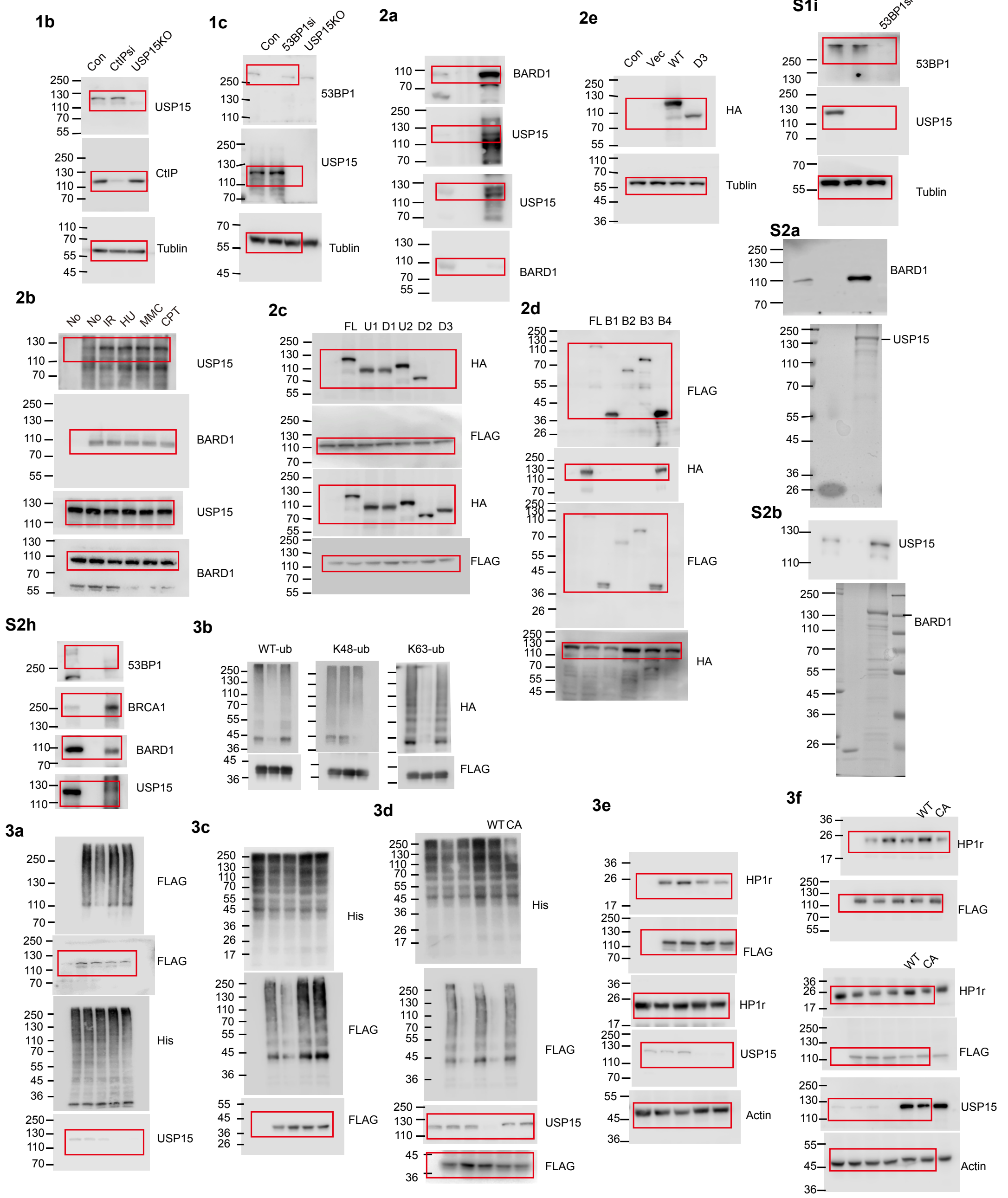

Supplementary Fig. 9 Full blots related to Fig. 3, Fig. 4, Fig. S3, Fig. S4.

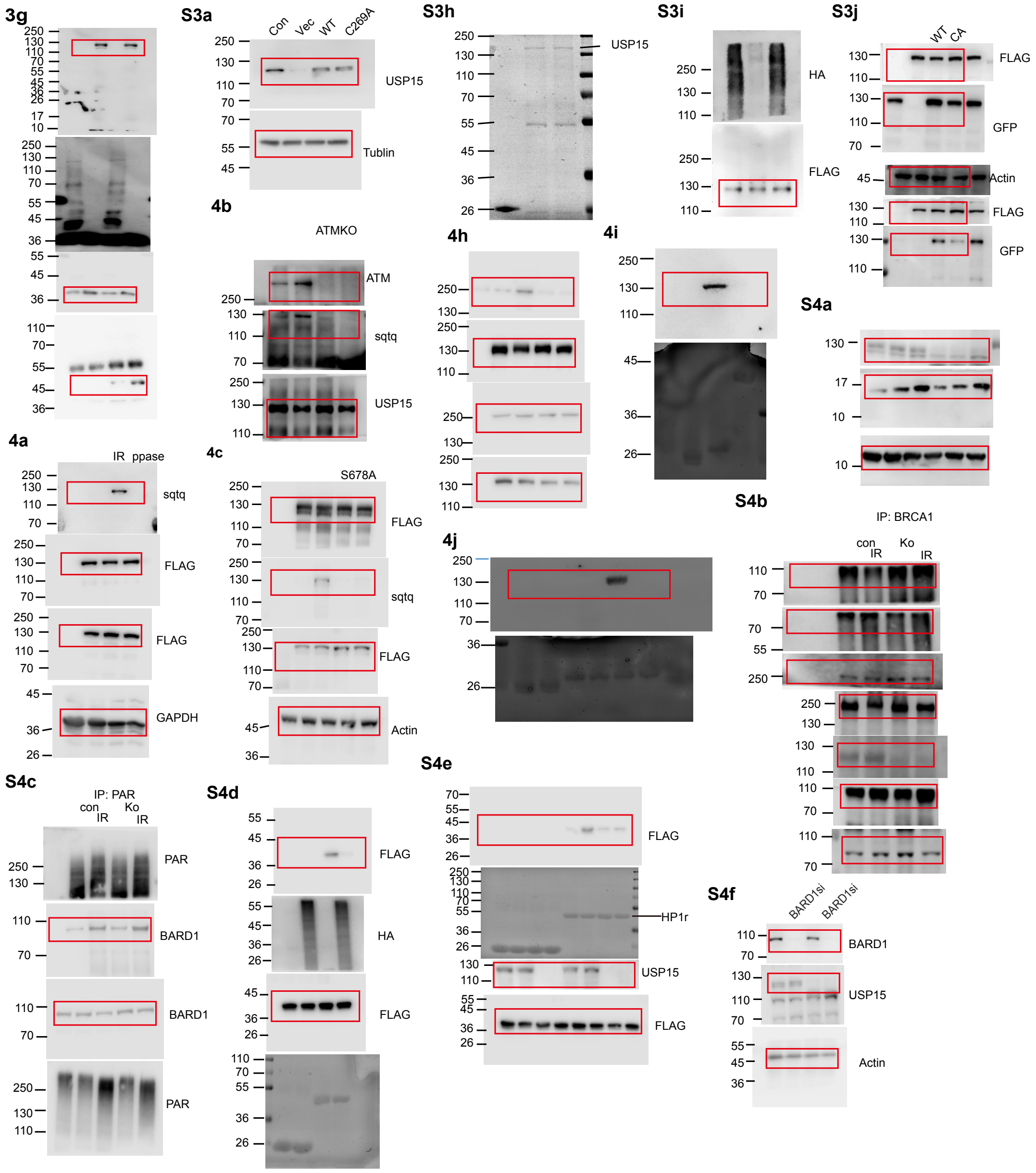

Supplementary Fig. 10 Full blots related to Fig. S4, Fig. 5, Fig. 6, Fig. 7 and Fig. S5, Fig. S6.

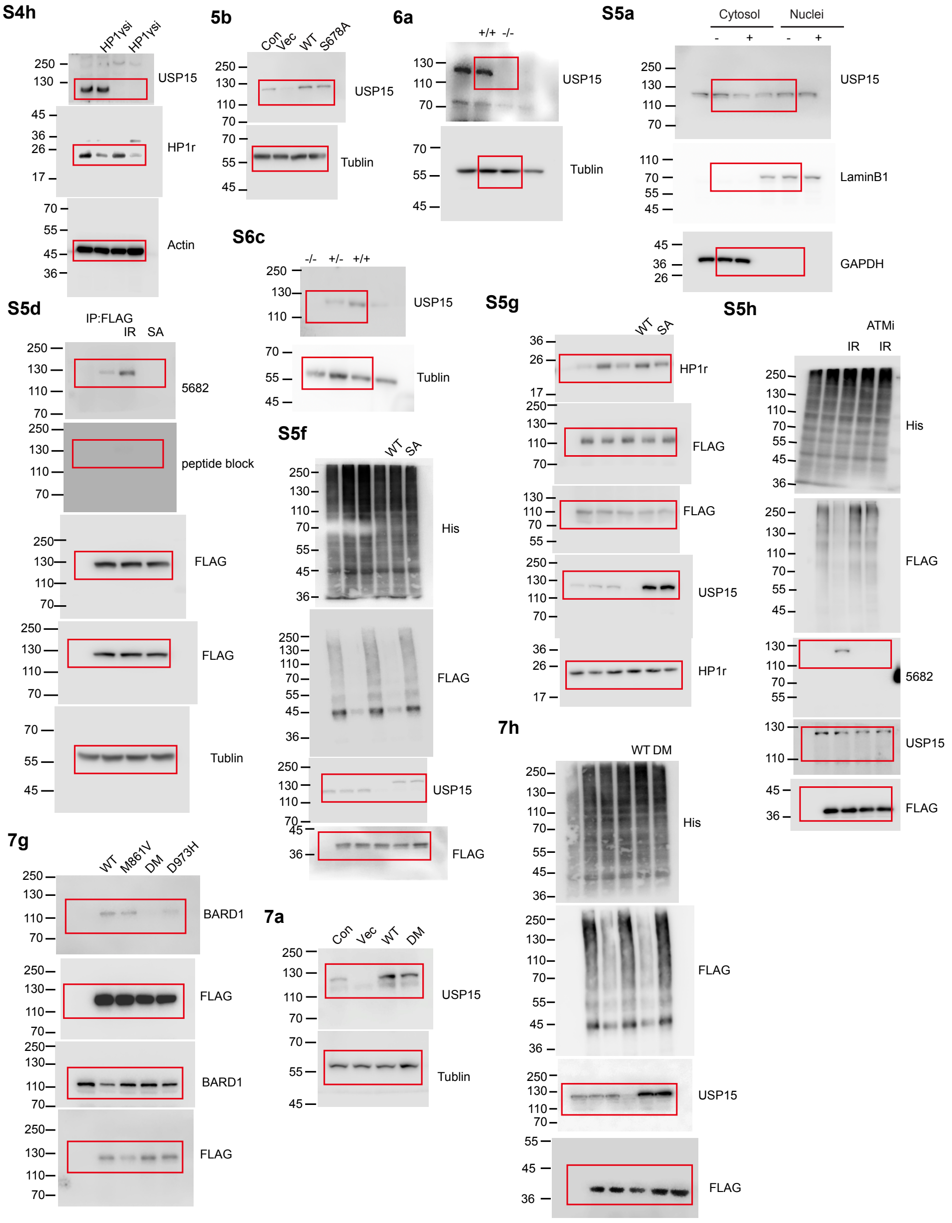

Supplement: Supplementary file 1 — Supplementary Information [file 41467_2019_9232_MOESM1_ESM.pdf]
