# Supplementary material for: The deubiquitylating enzyme USP15 regulates homologous recombination repair and cancer cell response to PARP inhibitors
Source: Nat Commun. 2019 Mar 15;10:1224. doi: 10.1038/s41467-019-09232-8 (PMC6420636; doi:10.1038/s41467-019-09232-8)
Supplement: Supplementary file 3 — Reporting Summary [file 41467_2019_9232_MOESM3_ESM.pdf]

nature research

Corresponding author(s): Huadong Pei
Last updated by author(s): Jan 27, 2019

Reporting Summary

Nature Research wishes to improve the reproducibility of the work that we publish. This form provides structure for consistency and transparency in reporting. For further information on Nature Research policies, see Authors & Referees and the Editorial Policy Checklist.

Statistics

For all statistical analyses, confirm that the following items are present in the figure legend, table legend, main text, or Methods section.

n/a	Confirmed	
☐	☒	The exact sample size (n) for each experimental group/condition, given as a discrete number and unit of measurement
☐	☒	A statement on whether measurements were taken from distinct samples or whether the same sample was measured repeatedly
☐	☒	The statistical test(s) used AND whether they are one- or two-sided<br>*Only common tests should be described solely by name; describe more complex techniques in the Methods section.*
☒	☐	A description of all covariates tested
☐	☒	A description of any assumptions or corrections, such as tests of normality and adjustment for multiple comparisons
☐	☒	A full description of the statistical parameters including central tendency (e.g. means) or other basic estimates (e.g. regression coefficient) AND variation (e.g. standard deviation) or associated estimates of uncertainty (e.g. confidence intervals)
☒	☐	For null hypothesis testing, the test statistic (e.g. F, t, r) with confidence intervals, effect sizes, degrees of freedom and P value noted<br>*Give P values as exact values whenever suitable.*
☒	☐	For Bayesian analysis, information on the choice of priors and Markov chain Monte Carlo settings
☒	☐	For hierarchical and complex designs, identification of the appropriate level for tests and full reporting of outcomes
☒	☐	Estimates of effect sizes (e.g. Cohen's d, Pearson's r), indicating how they were calculated

Our web collection on statistics for biologists contains articles on many of the points above.

Software and code

Policy information about availability of computer code

Data collection	Nikon NIS software was used to acquire immunofluorescence images.<br>GE healthcare's ImageQuant LAS 500 was used to acquire immunoblot data.
Data analysis	ImageJ 1.43u was used for image analysis.<br>Microsoft Excel and GraphPad Prism 6 were used for statistical analysis.<br>Nikon NIS software was used for fluorescence image processing.

For manuscripts utilizing custom algorithms or software that are central to the research but not yet described in published literature, software must be made available to editors/reviewers. We strongly encourage code deposition in a community repository (e.g. GitHub). See the Nature Research guidelines for submitting code & software for further information.

Data

Policy information about availability of data

All manuscripts must include a data availability statement. This statement should provide the following information, where applicable:

- Accession codes, unique identifiers, or web links for publicly available datasets
- A list of figures that have associated raw data
- A description of any restrictions on data availability

The authors declare that the data support the findings of this study are available within the article and supplementary information.

Field-specific reporting

Please select the one below that is the best fit for your research. If you are not sure, read the appropriate sections before making your selection.

☒ Life sciences ☐ Behavioural & social sciences ☐ Ecological, evolutionary & environmental sciences

For a reference copy of the document with all sections, see nature.com/documents/nr-reporting-summary-flat.pdf

Life sciences study design

All studies must disclose on these points even when the disclosure is negative.

Sample size	No statistical methods were used to predetermine the sample sizes. Chosen sample sizes were sufficient to acquire statistical significance between samples in all experiments.
Data exclusions	No samples or animals were excluded from the analysis.
Replication	Data were collected from three independent experiments.
Randomization	Animals or cells were randomly allocated into experimental groups prior to treatment.
Blinding	The investigators were not blinded to the group allocation and data collection.

Reporting for specific materials, systems and methods

We require information from authors about some types of materials, experimental systems and methods used in many studies. Here, indicate whether each material, system or method listed is relevant to your study. If you are not sure if a list item applies to your research, read the appropriate section before selecting a response.

Materials & experimental systems

n/a	Involved in the study	
☐	☒	Antibodies
☐	☒	Eukaryotic cell lines
☒	☐	Palaeontology
☐	☒	Animals and other organisms
☒	☐	Human research participants
☒	☐	Clinical data

Methods

n/a	Involved in the study	
☒	☐	ChIP-seq
☐	☒	Flow cytometry
☒	☐	MRI-based neuroimaging

Antibodies

Antibodies used	Antibody used in this study were as follows: Anti-USP15(A300-923A, WB: 1:1000), Anti-BARD1(A300-263A, WB: 1:1000; IF: 1:200), Anti-RAP80(A300-764A, WB: 1:1000; IF: 1:100) were purchased from Bethyl Laboratory. Anti-HP1γ（MABE656, WB: 1:2000）, Mouse anti-γ-H2AX(05-636, IF: 1:500), Mouse anti-MDC1 (05-1572, IF: 1:300), Anti-FK2 (04-263, IF: 1:200) were purchased from Millipore. Anti-RPA 70(#2267, IF: 1:200), Rabbit Anti-γ-H2AX(#9718, IF: 1:400) and Rabbit anti-HA (#3724, WB: 1:2500; IF: 1:300) were from Cell signaling Technology. Anti-Rad51(GTX100469, IF: 1:200) were from Genetex. Anti-BRCA1(D9, IF:1:50) and Anti-His tag (sc8036, WB: 1:1000) were from Santa Cruz. Anti-Poly PAR(4336-BPC-100, WB: 1:1000) were from Trevigon. Anti-53BP1 (NB100-304, WB: 1:1000; IF: 1:300) were from Novus Biologicals. Anti-RNF8 (14112-1-AP, IF: 1:100) were from Proteintech. Rabbit anti-FLAG (F7425, WB: 1:2500) and Mouse anti-FLAG (F3165, WB: 1:2500) were from Sigma Aldrich. Mouse anti-HA (901501, WB: 1:2500; IF: 1:100) were from Biolegend.
Validation	Commercial Primary antibodies used in this study were validated by the manufacturers. Phospho-specific USP15 antibody were validated within the Supplementary Information.

Eukaryotic cell lines

Policy information about cell lines

Cell line source(s)	HEK293T (ATCC, CRL-11268), U2OS (ATCC, HTB-96), MCF7 (ATCC, HTB-22) .
Authentication	Cell line authentication were performed by ATCC.

Mycoplasma contamination	All the cell lines were tested negative for mycoplasma.
Commonly misidentified lines (See ICLAC register)	No misidentified cell lines were used in this study.

Animals and other organisms

Policy information about studies involving animals; ARRIVE guidelines recommended for reporting animal research

Laboratory animals	USP15 knockout mice were generated from C57BL/6J strains.
Wild animals	No wild animals were used in this study.
Field-collected samples	No field-collected samples were used in this study.
Ethics oversight	The animal protocol was reviewed and approved by the Animal Care and Use Committee of the Beijing Proteome Research Center.

Note that full information on the approval of the study protocol must also be provided in the manuscript.

Flow Cytometry

Plots

Confirm that:

- [ ] The axis labels state the marker and fluorochrome used (e.g. CD4-FITC).
- [ ] The axis scales are clearly visible. Include numbers along axes only for bottom left plot of group (a 'group' is an analysis of identical markers).
- [ ] All plots are contour plots with outliers or pseudocolor plots.
- [ ] A numerical value for number of cells or percentage (with statistics) is provided.

Methodology

Sample preparation	U2OS-DR-GFP or U2OS-EJ5-GFP cells were trypsinized and resuspended in PBS solution.
Instrument	BD Influx Cell Sorter
Software	Flow jo
Cell population abundance	10million per ml
Gating strategy	510-40nm for GFP positive cells/610-20nm for mcherry positive cells.

- [ ] Tick this box to confirm that a figure exemplifying the gating strategy is provided in the Supplementary Information.
